# Oxidative Stress in Human Atherothrombosis: Sources, Markers and Therapeutic Targets

**DOI:** 10.3390/ijms18112315

**Published:** 2017-11-03

**Authors:** Jose Luis Martin-Ventura, Raquel Rodrigues-Diez, Diego Martinez-Lopez, Mercedes Salaices, Luis Miguel Blanco-Colio, Ana M. Briones

**Affiliations:** 1 Vascular Research Lab, FIIS-Fundación Jiménez Díaz-Autonoma University, 28040 Madrid, Spain; diegomartilo@hotmail.com (D.M.-L.); lblanco@fjd.es (L.M.B.-C.); 2 Centro de Investigación Biomédica en Red de Enfermedades Cardiovasculares (CIBERCV), Spain; 3 Departamento de Farmacología, Facultad de Medicina, Universidad Autónoma de Madrid, 28029 Madrid, Spain; raquel.rodrigues@inv.uam.es (R.R.-D.); mercedes.salaices@uam.es (M.S.); 4 Instituto de Investigación Hospital Universitario La Paz (IdiPAZ), 28046 Madrid, Spain

**Keywords:** atherothrombosis, oxidative stress, lipids/lipoprotein oxidation, biomarkers

## Abstract

Atherothrombosis remains one of the main causes of morbidity and mortality worldwide. The underlying pathology is a chronic pathological vascular remodeling of the arterial wall involving several pathways, including oxidative stress. Cellular and animal studies have provided compelling evidence of the direct role of oxidative stress in atherothrombosis, but such a relationship is not clearly established in humans and, to date, clinical trials on the possible beneficial effects of antioxidant therapy have provided equivocal results. Nicotinamide adenine dinucleotide phosphate (NADPH) oxidase is one of the main sources of reactive oxygen species (ROS) in human atherothrombosis. Moreover, leukocyte-derived myeloperoxidase (MPO) and red blood cell-derived iron could be involved in the oxidative modification of lipids/lipoproteins (LDL/HDL) in the arterial wall. Interestingly, oxidized lipoproteins, and antioxidants, have been analyzed as potential markers of oxidative stress in the plasma of patients with atherothrombosis. In this review, we will revise sources of ROS, focusing on NADPH oxidase, but also on MPO and iron. We will also discuss the impact of these oxidative systems on LDL and HDL, as well as the value of these modified lipoproteins as circulating markers of oxidative stress in atherothrombosis. We will finish by reviewing some antioxidant systems and compounds as therapeutic strategies to prevent pathological vascular remodeling.

## 1. Introduction

Atherothrombosis is the main cause of death in developed countries. The main feature underlying atherothrombosis is a chronic pathological remodeling of the vascular wall, characterized by lipid deposition, oxidative stress, immune-inflammatory and proliferative responses, along with proteolysis, neo-angiogenesis, apoptosis, calcification and fibrosis [1,2]. Reactive oxygen species (ROS) are considered crucial mediators of vascular homeostasis and pathogenesis in vascular diseases. Low levels of ROS are essential for the regulation of multiple cellular processes and signaling pathways, whereas uncontrolled ROS production, as occurs in several vascular diseases including atherosclerosis or abdominal aortic aneurysm (AAA), results in exacerbated oxidative stress that damages vascular cells through a myriad of processes [3,4,5,6,7,8].

Known risk factors for atherothrombosis include increased systemic low-density lipoprotein (LDL) and reduced high-density lipoprotein (HDL) cholesterol levels. This systemic alteration of lipoprotein particles is accompanied by the increased lipoprotein retention observed during the earlier stages of the development of the vessel wall remodeling. LDLs are highly susceptible to being modified by the oxidative milieu found inside the vascular wall. In fact, the “oxidative modification hypothesis of atherosclerosis” [9] was based on the evidence that modified oxidized LDLs are retained in atherosclerotic plaques and their uptake by scavenger receptors on phagocytes lead to foam cell formation. In addition, oxidative stress could also modify other lipoproteins (e.g., HDL) or other molecules involved in different initial processes associated with vessel wall remodeling (e.g., nitric oxide-related endothelial dysfunction). However, the precise sources of oxidative stress in these initial stages are not completely defined.

In the more advanced stages, intraplaque hemorrhages in complicated atherothrombotic disease [10] and intraluminal thrombus (ILT) in AAA [11] both lead to clinical complications due to arterial wall rupture, involving intimal cap rupture in complicated atherothrombotic plaques and medial and adventitial rupture in AAA. No matter where there is intraplaque or intraluminal localization, hemorrhages and/or thrombi involve trapping of red blood cells (RBCs), leukocytes and activating platelets. In this context, RBC-derived, iron-rich heme group and leukocyte-derived oxidants (e.g., NADPH-dependent ROS and myeloperoxidase-MPO-), are the main sources of oxidative stress and are able to modify lipids, proteins and DNA, which leads to the progression of atherothrombotic pathology towards clinical events [12].

In the present review, we will summarize the molecules involved in redox imbalance in human atherothrombosis, highlighting the functional consequences of oxidative stress mainly in lipoproteins, due to their key role in vascular diseases. Moreover, we will describe studies by analyzing the potential use of some biomarkers of redox imbalance, as well as its potential therapeutic value, in these pathologies.

## 2. Generation and Elimination of ROS

ROS are reactive derivatives of oxygen metabolism. These include molecules with unpaired electrons, also termed free radicals such as superoxide anion (O_2_^−^) and hydroxyl radical (OH), which are highly unstable and have short half-lives. Non-radical ROS include more stable molecules with longer half-lives such as hydrogen peroxide (H_2_O_2_), peroxynitrite (ONOO^−^) and hypochlorous acid (HOCl) [13]. The majority of O_2_^−^ generated is rapidly converted to H_2_O_2_, which, in contrast to O_2_^−^, penetrates cell membranes easily, and functions as a second messenger that activates multiple signaling pathways.

**Figure 1:**
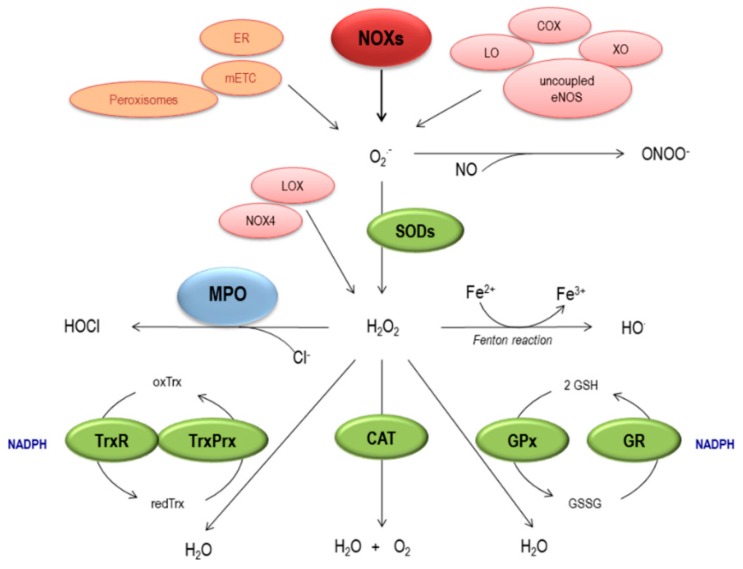
Generation and elimination of ROS. Enzymatic systems (in red) including NADPH oxidases (NOXs), Xanthine Oxidase (XO), Lipoxygenase (LO), Cyclooxygenase (COX) and uncoupled eNOS produce O_2_^−^ that can also be generated non-enzymatically (in orange) by the mitochondrial electron transport chain (mETC), the endoplasmic reticulum (ER) and peroxisomes. O_2_^−^ is then transformed into H_2_O_2_ spontaneously or through superoxide dismutases (SODs) or can be synthesized directly by NOX-4 or as a by-product of lysyl oxidase (LOX). O_2_^−^ can rapidly react with NO leading to the formation of ONOO^−^. H_2_O_2_ can be then converted into more reactive molecules, including hydroxyl radical (OH^−^) by Fenton reaction or into HOCl by myeloperoxidase (MPO). Furthermore, H_2_O_2_ can also be transformed into H_2_O by catalase (CAT) or by the glutathione peroxidase (GPx)/gluthathione reductase (GR) and the thioredoxin (Trx)/peroxiredoxin (PRx) systems. TrxR: thioredoxin reductase; TrxPrx: thioredoxin peroxidase.

O_2_^−^ is formed by the univalent reduction of molecular oxygen. This process is mediated by different enzymatic systems including NADPH oxidases (NOX), xanthine oxidase, lipoxygenase, cyclooxygenase, CYP450 isoforms, monoxygenases and uncoupled endothelial NO synthase (eNOS). O_2_^−^ can also be generated non-enzymatically by the mitochondrial electron transport chain, the endoplasmic reticulum (ER), and peroxisomes (Figure 1) [13,14,15,16]. O_2_^−^ can be converted into H_2_O_2_ spontaneously or by the superoxide dismutases (SOD) enzymes: cytosolic Cu/Zn-SOD (SOD1), mitochondrial Mn-SOD (SOD2) and extracellular EC-SOD (SOD3). Moreover, some types of NOX including NOX-4 and dual oxidases (DUOX)-1 and -2, can directly produce H_2_O_2_ [16], which can also be synthesized as a by-product of different enzymes including some which are important in cardiovascular diseases (CVD) such as lysil oxidase [17,18] (Figure 1).

In the presence of reduced transition metals (e.g., ferrous or cuprous ions), H_2_O_2_ can be converted into the highly reactive OH that damages different macromolecules including lipids, proteins and DNA. Alternatively, H_2_O_2_ may be converted into water by the enzyme catalase or glutathione peroxidase-1 that catalyzes the reduction of H_2_O_2_ using reduced glutathione (GSH) as an electron donor. GSH is transformed into glutathione disulfide (GSSG) by glutathione peroxidase, which can then be converted back to GSH by glutathione reductase in an NADPH-consuming process (Figure 1). The thioredoxin (TRX) system, in which thiol-dependent peroxidases (peroxiredoxins, PRDX) are provided with electrons to remove reactive oxygen and nitrogen species rapidly, is also an important H_2_O_2_ detoxifying system [19].

Myeloperoxidase (MPO) is a well-known enzyme, mainly released by activated neutrophils, characterized by powerful pro-oxidative and pro-inflammatory properties. MPO is a heme peroxidase that produces HOCl in the reaction between H_2_O_2_ and chloride ions. These mediators are not only important for the antimicrobial activities of the innate immune system but also contribute to immune inflammatory diseases, including atherosclerosis and AAA [6,20] (see Section 3.2).

Other important ROS/reactive nitrogen species (RNS) is peroxynitrite (ONOO^−^) which is formed by the reaction of NO with O_2_^−^. RNS produce post-translational modifications of proteins, nitrative stress and different modifications such as tyrosine nitration. Moreover, not only ONOO^−^ but also MPO pathways have been involved in protein nitration [20].

Increased levels of ROS activate nuclear factor (erythroid-derived 2)-like 2 (Nrf2), a master regulator of the antioxidant response, which is activated to counteract oxidative stress. Nrf2 controls the expression of about 250 genes including those encoding antioxidant enzymes such as those involved in glutathione and TRX systems, SOD, catalase, and hemoxygenase-1, among many others [21]. In addition to enzymatic degradation of ROS, various low-molecule compounds can directly react with ROS. These molecules can be endogenously synthesized or obtained from diet and include vitamins C and E, uric acid, glutathione, flavonoids and thiols, among others [3].

The misbalance between ROS generation and elimination determines oxidative stress. It is now accepted that oxidative stress responses are involved in many cellular and tissue processes in relation to CVD and its risk factors. In fact, all established cardiovascular risk factors such as hypercholesterolemia, hypertension, diabetes mellitus, and smoking enhance ROS generation. The ROS-modulated processes include proliferation and migration of vascular smooth muscle cells (VSMC), endothelial dysfunction with diminished NO availability and increased vasoconstriction, and increased production of isoprostanes. These isoprostanes are eicosanoids derived from nonenzymatic oxidation of arachidonic acid via the interaction with ROS and they cause artery vasoconstriction via TP receptors, VSMC proliferation and platelet aggregation. Moreover, endothelial activation with the expression of adhesion molecules, recruitment of inflammatory cells, lipid oxidation, platelet aggregation, activation of metalloproteinases and altered extracellular matrix deposition, are also activated by ROS [3,4,5,6,7]. All cells in the vascular wall including VSMC, endothelial cells and adventitial cells, together with circulating cells (such as platelets and RBC) are able to generate ROS. Moreover, inflammatory cell infiltration is now recognized as a potential source of ROS in different CVD including atherosclerosis and AAA. Most of the vast information available on the role of oxidative stress in CVD has been obtained from animal models and excellent reviews covering these issues are already available [7,8,22,23,24,25,26,27]. For the sake of clarity, we now discuss in depth the possible role of oxidative stress in atherothrombosis in the human context.

## 3. Sources of Oxidative Stress in Human Vascular Diseases

**Figure 2:**
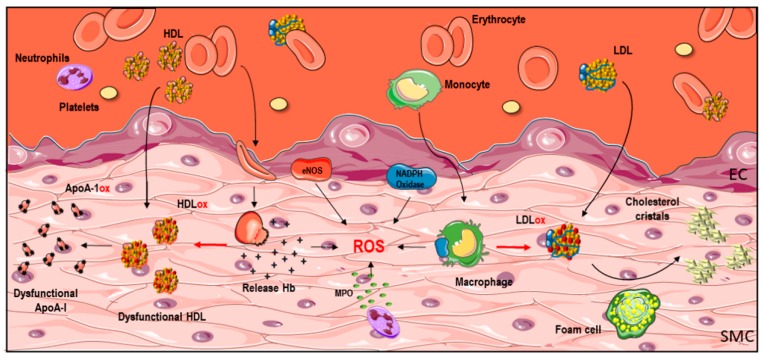
Sources of oxidative stress in the vascular wall. The oxidative process inside the pathological vascular wall is the result of the interaction of lipids/lipoproteins and reactive oxygen species (ROS) derived from infiltrating (red blood cells-RBC, platelets, leukocytes-neutrophils and monocytes) and resident (endothelial cells-EC- and smooth muscle cells-SMC-) cells. LDL, low-density lipoproteins; HDL, high-density lipoproteins; ApoA1, apolipoprotein A1; MPO, myeloperoxidase; Hb, hemoglobin; eNOS, endothelial NO synthase. Some graphical elements from this figure were adapted from Servier Medical Art Powerpoint image bank at http://smart.servier.com/.

In this section, we will focus on NADPH oxidase as a master of ROS production, but also on leukocyte-derived MPO and in RBC-derived iron that have been linked to lipid/lipoprotein oxidation in humans (Figure 2).

### 3.1. NADPH Oxidase

The NADPH oxidase system is the main source of ROS in the vessel wall and is present in endothelial cells, VSMC, adventitial fibroblasts, and infiltrating monocytes/macrophages. The structure and function of NADPH oxidase in physiological and cardiovascular pathological conditions have been extensively reviewed [5,6,14,16,28,29,30]. In contrast to the rest of the ROS-producing enzymes that produce ROS as a by-product of their activity, the main catalytic function of NADPH oxidases is the generation of ROS. NADPH oxidase isoforms in mammals have a catalytic subunit called NOX (NOX-1-5) or DUOX (DUOX-1-2) and up to seven regulatory subunits, leading to the formation of seven NADPH oxidase isoforms. NOX-1, NOX-2, NOX-4 and NOX-5 are expressed in the cardiovascular system. The classic NOX, NOX-2, was initially found and characterized in leukocytes. Cytosolic adaptor proteins called “NOX organizers” (p47phox or NOXO1 and p40phox) and “NOX activators” (p67phox or NOXA1) that bind GTP-Rac and affect the flow of electrons, regulate the activity of NOX-1, NOX-2 and NOX-3. When the p22phox component binds with NOX-1-4, a stable heterodimeric complex and then the active oxidase are formed [5,6,14,16,28,29,30]. NOX isoforms are variably expressed in vascular cells with some of them coexisting in the same cell type, suggesting different cell functions for each NOX. Thus, endothelial cells express NOX-1, NOX-2, NOX-4 and NOX-5, the latter being expressed only in humans; VSMCs mainly express NOX-1, NOX-4 and NOX-5; and adventitial cells mainly express NOX-2 and NOX-4 [5,6,14,16,28,29,30]. As mentioned, NOX-2 is mainly expressed in phagocytes (neutrophils and macrophages) but platelets also express NOX-2 where it has a central role in generating O_2_^−^ [6,31].

Many studies in animal models have demonstrated a key role of vascular and phagocytic NADPH oxidase isoforms in the development of vascular diseases and to some degree these observations have also been extended to humans. Vascular production of O_2_^−^ increases as a consequence of risk factors for atherosclerosis [32]. Atherosclerotic lesions contain abundant p22phox and NOX-2 (also termed gp91phox) that correlated with the severity of atherosclerosis [33,34] where p22phox is located in adventitial fibroblasts, VSMC, macrophages in the neointima and media, and in endothelial cells [35]. Importantly, clinical and experimental studies support the role of NOX-2 expressed in platelets in the atherothombotic process [7,8] by mechanisms that include the expression of the CD40 ligand, a protein with pro-inflammatory and prothrombotic properties on interaction with its receptor CD40 thereby modulating platelet function [6,31]. The role of NOX-1 in atherogenesis remains controversial since NOX-1 was undetected or had very low expression in human lesions [33,34]. Interestingly, NOX-1 upregulation was demonstrated in plaques from patients with cardiovascular events or established diabetes mellitus [36]. In contrast, NOX-4 was found exclusively in non-phagocytic cells with NOX-4 being highest in stage IV atherosclerosis and dramatically decreased in the most complicated plaques that are characterized by fibrosis and a reduction in intimal VSMC [34]. Another study also showed that NOX-4 mRNA levels were reduced in plaques from patients with cardiovascular events or established diabetes mellitus which was found that, together with experimental studies, pointed to a possible role of NOX-4 as a negative modulator of inflammation and remodeling to convey atheroprotection [36]. Finally, NOX-5 was found to be upregulated in atherosclerosis in the endothelium in the early lesions and in VSMC in the advanced coronary lesions [37] and more recently, NOX-5 was found in human monocytes and macrophages and in macrophage-rich areas within human carotid artery atherosclerotic plaques [38]. However, the fact that NOX-5 is only expressed in humans has slowed down the progress in the elucidation of the impact of NOX-5 in atherosclerosis.

Analysis of tissue from patients undergoing bypass surgery revealed that, besides changing NOX isoforms, diabetes is characterized by increased expression of p22phox, p47phox, and p67phox compared with non-diabetics [39]. Moreover, NADPH oxidase activity in peripheral blood mononuclear cells positively correlated with carotid intima-media thickness, a surrogate marker of atherosclerosis, in asymptomatic subjects [40]. Increased expression and activity of NADPH oxidases are also important mechanisms underlying oxidative stress in human AAA [41]. Specifically, mRNA levels of p22phox, NOX-2 and NOX-5 were significantly increased in AAAs while NOX-4 mRNA expression was lower [41]. Notably, although human studies clearly suggest a role for increased oxidative stress in atherothrombosis, the specific cell responsible remains elusive.

In humans, direct evidence of the relationship between ROS and atherothrombosis mainly originate from studies in patients with NOX-2 loss of function by genetic NOX-2 or p47phox deficiencies (chronic granulomatous disease). These patients show increased flow mediated dilation and therefore NO-induced vasodilation, and diminished carotid intima-media thickness, two surrogate markers of atherosclerosis [7,8,42,43]. Interestingly, reduced carotid but not coronary artery atherosclerosis was observed in patients with chronic granulomatous disease suggesting that NOX2-related mechanisms may play a lesser role in coronary atherosclerosis than in other arterial beds [44]. In the same line of evidence, patients with the C242T polymorphism of the p22phox subunit (associated with lower oxidative stress) [45], had less cardiovascular death, myocardial infarction and re-vascularization compared with those carrying the wild-type allele [46], demonstrating that the 242T allele was a predictor of lower risk of recurrence of cardiovascular events in high-risk patients.

### 3.2. MPO

MPO is a hemoprotein mainly released by activated leukocytes that catalyzes the reaction between H_2_O_2_ and chloride ions to produce HOCl as the primary oxidant. MPO-derived oxidants generate a footprint of specific (e.g., 3-Chlorotyrosine, 3-Cl-Tyr) and nonspecific (e.g., protein carbonyls and 3-nitrotyrosine modifications) oxidation products. Moreover, MPO may serve as a source of free iron through a mechanism that involves heme depletion [47] and MPO has been also implicated in lipoprotein oxidation in vivo [48]. It was long established that MPO works as an NO-oxidase, consuming NO to lead to impaired endothelial relaxation [49]. Previous studies have shown that catalytically active MPO and its oxidative species are present in human atherothrombotic tissues [50,51,52,53]. Moreover, plasma MPO levels are increased in atherosclerotic and AAA patients [53,54]. It should be noted that MPO is a potent predictor of cardiovascular events in patients with chest pain [55] and MPO levels are a significantly better predictor of major adverse cardiovascular events than NT-proBNP levels in patients with ST-segment elevation in myocardial infarction who are treated with primary percutaneous coronary intervention [56]. More recently, increased MPO indexed to HDL particle concentration at baseline is associated with increased risk of incident cardiovascular events in a population initially free of CVD [57].

### 3.3. Iron

Iron plays crucial roles in cell proliferation and metabolism by serving as a functional constituent of various enzymes, normally associated to the hemo group. The main iron pool in the body is found within the hemoglobin (Hb) of RBCs, which, after a mean half-life of 120 days, are taken up by resident macrophages by erythrophagocytosis. However, free iron is toxic through the generation of ROS via the Fenton reaction. Iron could be released due to microvessel rupture in atherosclerotic plaques [10] or after RBC lysis within the intraluminal thrombus of AAA [11]. Iron was observed in advanced carotid atherosclerotic plaques [58]. Moreover, it has been recently described that RBC efferocytosis by the arterial wall promotes oxidation in early-stage human atheroma [59]. In this respect, the role of iron in the pathogenesis of atherosclerosis was originally associated with its ability to catalyze the oxidation of lipoproteins [60], but potential novel mechanisms by which iron could modulate atherogenesis have been later described [61]. More recently, Sawada et al. described that iron was accumulating in human AAA walls compared with non-AAA walls and the extent of the iron-accumulated area positively correlated with that of the area of 8-hydroxy-2′-deoxyguanosine expression [62]. A long time ago, iron was proposed as a cardiovascular risk factor, suggesting that the lower incidence of CVD in premenopausal women could be explained by the lower body iron stores [63]. We also described local iron retention and altered iron recycling associated with high hepcidin and low transferrin systemic concentrations in AAA patients, potentially leading to reduced circulating Hb levels [64]. Moreover, low Hb levels were associated with AAA progression. In this respect, anemia has been associated with several chronic (inflammatory) diseases, including CVD, probably related to the diversion of iron recycling [65,66].

## 4. Markers of Oxidative Stress in Human Vascular Diseases

A biomarker is a marker reflecting or integrating one or several biological activities. In the case of biomarkers of oxidative stress, we will refer to both pro- and antioxidant biomarkers. Such markers may be any detectable and quantifiable molecules including proteins, peptides, lipids, nucleic acids, etc. Many studies have reported oxidative stress markers in tissues and plasma of patients with atherothrombosis. However, although markers assessing the oxidation of phospholipid and protein components of LDL were among the first to be developed, clinical trials including cross-sectional and retrospective and prospective studies provided equivocal results [6]. Among the reasons explaining these conflicting results, methodological issues have been highlighted and it is beyond the scope of this article to review this aspect in detail and the reader is referred to excellent reviews on this aspect [6,20,67]. In any case, although promising advances in this field are being carried out in the last years, it is still premature to unequivocally affirm the clinical validity of a specific oxidative stress biomarker for the management of patients with higher risk of CVD [6].

### 4.1. Oxidized LDL

Low-density lipoprotein (LDL) is the main player in cholesterol transport to the cells and high concentration of LDL is a well-established cardiovascular risk factor [68]. LDL has been associated to atherosclerosis development as in the subendothelial space, LDL becomes modified by either aggregation, acetylation and/or oxidation. Modified LDL induces endothelial injury, increases the expression of adhesion molecules, favoring monocyte adhesion and its differentiation to macrophages [69]. Moreover, oxidized LDL stimulates platelet aggregation and inhibits endothelial NO synthase expression/activity, promoting atherogenesis. Therefore, modified LDL is a main mediator inducing vascular damage and atherosclerotic disease development.

Oxidized LDLs are mainly present in ceroids that can be formed within the cell and are similar to cholesterol crystals [70]. Ceroids are autofluorescent, insoluble and sudanophilic polymers composed of aggregated proteins entrapping lipids. Iron deposits, Hb and MPO colocalized with ceroids within cells and tissues such as atherosclerotic plaques and AAA [71,72,73]. After the initial finding of the presence of ceroid/lipofuscin and of peroxidized lipids in atherosclerotic lesions [74,75], it was discovered that oxLDLs are present in atherosclerotic lesions [76]. Oxidation of LDL can be carried out by, among others, transition metals, Hb, lipoxygenases, and ROS generated by vascular cells or phagocytes. Interestingly, oxidation of LDL by NOX-2 containing platelets may represent another mechanism through which NOX-2 activates platelets in a self-perpetuating mechanism [7,8]. HOCl can modify LDLs at the lipid and the protein moieties in vitro and/or in vivo [77]. Malondialdehyde (MDA), a lipid peroxide product released by oxidation from prostanoid metabolism, reacts with the positively charged epsilonamino group of apo B-100 protein lysyl residues, a constituent of the LDL molecular complex [78]. MDA-modified LDLs induce lipid accumulation in macrophages [79]. Circulating MDA-LDL levels has been proposed as a marker of oxidative stress in atherosclerotic CVD [80,81,82] and clinical studies have demonstrated that MDA-LDL levels are associated with the severity of coronary artery disease [83], coronary plaque vulnerability [84] and adverse clinical outcomes after percutaneous coronary intervention with drug eluting stents [85].

Oxidized LDL is immunogenic and the oxidative modifications of apolipoprotein B-100 resulted in the formation of neoepitopes. Oxidation-specific epitopes (OSE) may be indirectly reflected by the presence of circulating antibodies and immune complexes. These are often measured as IgG and IgM autoantibodies to MDA-LDL and apoB-immune complexes [86]. These biomarkers can predict CVD and associated events [87]. In atherosclerotic CVD, IgG and IgM titters to OSE such as MDA-LDL, were predictive of recurrent events in a prospective study with a 15-year follow-up [88]. In general, high levels of IgM OSE biomarkers predict lower risk, consistent with their potential protective function as natural antibodies, and higher levels of IgG biomarkers predict a higher risk, consistent with their general properties of being acquired.

### 4.2. Oxidized HDL

Although most of the work related to the oxidation hypothesis of atherosclerosis has been performed on LDL, there is also evidence that oxidation of other lipoproteins, such as HDL, could also take place during vascular remodeling. In this respect, it is important to note that the majority of cholesterylester hydroperoxides are associated with HDLs rather than LDLs [89]. HDL is the responsible for the reverse cholesterol transport, which is the transport of excess cholesterol from the peripheral tissues to the liver for its elimination in feces and bile [90]. HDL is atheroprotective by several ways; one of them is cholesterol efflux from macrophages, controlling the accumulation of foam cells and atherosclerosis development. Beyond the role of HDL in reverse transport of cholesterol, the particle is protective through other functions such as anti-inflammatory, antioxidant, antithrombotic, anti-fibrotic and vasoprotective properties, protection against lipopolysaccharide and promotion of NO production [91,92].

Epidemiologically, an inverse relationship between HDL cholesterol (HDLc) and cardiovascular risk has been clearly demonstrated [93]. Low HDLc levels are also associated with both AAA presence and progression [94,95,96]. However, HDLc-raising therapies do not result in cardiovascular risk reduction [97]. These negative results have led to a new HDL perception, where the “quality” or “functionality” is more relevant than just HDL plasma levels [98]. It is now fully accepted that in pathological states, such as the oxidative and pro-inflammatory environment present in atherothrombosis, HDL is remodeled, modifying the functionality of the particle. Among functional assays measuring HDL quality, it has been shown that decreased cholesterol efflux capacity is related to incident CVD and CV events [99,100]. In addition, HDL functionality is associated with its molecular (protein/lipid) composition [101]. Regarding AAA, it was previously demonstrated that HDL carries less alpha-1 antitrypsin and higher MPO levels, leading to dysfunctional HDL characterized by decreased antioxidant properties [73,102]. The functionality of the particles could also be derived by the presence of postranslationally-modified proteins. Among them, it has been previously demonstrated that ApoA1, the main constituent of HDLs, could be oxidatively modified, leading to dysfunctional HDLs [103,104,105,106,107]. Previous studies of ApoA1 from human aortic tissues revealed that ApoA1 in the human aorta was extensively oxidatively cross-linked and functionally impaired [108]. Moreover, elevated oxApoA1 levels in a large cohort of subjects presented to a cardiology clinic were associated with increased CVD risk [109]. Similarly, Yassine et al. demonstrated a significant increase in oxApoA1 in the HDL of participants with diabetes and CVD compared to participants without CVD [110].

### 4.3. Antioxidants

Paraoxonase 1 (PON1) hydrolyzes lipoprotein-associated peroxides and lactones. PON1 is mainly synthesized in the liver and in circulation it is associated with HDL. However, PON1 is not a fixed component of HDL since the enzyme could also exert its protective functions outside the lipoprotein environment. It has been demonstrated that HDL transfers PON1 to cell membranes to improve cellular resistance to oxidative stress [111,112]. Previous studies supported a role of PON1 in atheroprotection, through its ability to prevent lipid oxidation and limit atherosclerotic lesion development; moreover, low PON1 activity has been associated with different cardiovascular pathologies, including atherosclerosis and AAA [113,114,115,116,117]. In addition, human population studies have suggested an association of PON-1 polymorphisms with CVD [118].

As mentioned, catalase is one of the most active catalysts that decompose H_2_O_2_ at an extremely rapid rate and without consuming cellular reducing equivalents [119]. H_2_O_2_ itself is not very reactive; however, the danger of H_2_O_2_ comes from its ready conversion to hydroxyl radical by the interaction with a range of transition metal ions, of which the most important in vivo is probably iron. We have recently observed a decrease in catalase, along with SOD and thioredoxin (TRX) reductase, in polimorphonuclear cells (PMNs) of AAA patients compared to controls, which suggest a global decrease in antioxidant enzymes in PMNs under chronic pathological conditions [120]. In contrast, increased catalase immunostaining was shown in AAA tissue [120], which is similar to what is observed in atherosclerotic plaques [121].

Among protein thiol-disulfide oxidoreductases, TRX and PRDX have been widely associated with atherothrombosis. TRX is overexpressed in cells of the vascular wall, probably as a response to high oxidative stress [122]. In contrast, the truncated form, called TRX80, was associated with a pro-inflammatory status and increased atherosclerosis [123]. Moreover, increased expression of TRX has been observed in complicated human atherosclerotic plaques, associated with augmented ROS production and intraplaque hemorrhage [124,125]. Similarly, TRX reductase overexpression is observed in atherosclerotic plaques [126]. Different PRDX isoforms seem to modulate different cellular responses. PRDX1 diminishes leucocyte activation and adhesion to vascular endothelium. Moreover, PRDX-1 was observed in both VSMC and macrophages in human atherosclerotic plaques [127]. Similarly, PRDX-1 and -2 were detected in AAA tissue [128,129], probably as a response to increased oxidative stress [130]. TRX and PRX levels are elevated in plasma from atherothrombotic patients [131,132]. We reported an increase in serum TRX, but also PRX-1, from AAA patients compared with control subjects. Besides, TRX and PRX-1 correlates with AAA size and expansion rate, which suggests that TRX and PRX-1 could be good biomarkers of AAA evolution [128,133]. The increased levels of TRX-1/PRX-1 associated to disease have been suggested to represent a response to increased oxidative stress. In this regard, we recently observed that TRX-1/PRX-1 levels in plasma of asymptomatic subjects correlated with NADPH activity in peripheral blood mononuclear cells [127].

## 5. Antioxidants as a Potential Therapeutic Strategy to Prevent Pathological Vascular Remodeling

Based on the above findings, antioxidant therapy seems to be a promising alternative for the treatment of atherothrombosis and its associated complications. However, disappointing results have been obtained when comparing results obtained in animal models and in patients. Thus, different antioxidants have in general, prevented, slowed or even reversed atherosclerosis or AAA in animal models. In addition, these findings have been greatly reinforced by the fact that knockout mice on different ROS producing enzymes including NADPH oxidases, or transgenic mice overexpressing detoxifying enzymes including catalase, are partially protected against different processes involved in the atherothrombotic process (see below in this section). To date, no antioxidant drugs have proven effective in the treatment of atherothombotic complications in patients. The majority of trials evaluated the effects of vitamins (mainly vitamin C and E) or folic acid and showed negative results. Among potential explanations, it has been suggested that there were probable differences in oxidative stress in the patients and that they were not assessed for their “oxidative stress status” [6,134]. Other factors such as the type of vitamin given alone or in combination, administration with or without meals, concomitant use with other potential antioxidant drugs, lack of site-specificity or dosage and duration of antioxidant used have also been questioned [8,134]. Other potential antioxidant therapeutic approaches in humans are N-acetylcysteine, an antioxidant precursor of the synthesis of GSH, or nutritional supplements mainly included in the Mediterranean diet such as polyphenols present in extra virgin olive oil, chocolate, red wine or black and green tea, which in general seems to be associated with lower CVD (reviewed in detail by Violi et al. [8]). In this line of evidence, novel antiplatelet and antithrombotic therapies using different antioxidant compounds including flavonoid conjugates, isoquercetine or N-acetylcysteine are being tested [135,136,137]; however, the influence of their antioxidant activity to the antithrombotic and antiplatelet activities remains to be established.

Another interesting finding relies on the fact that many commonly used drugs for the treatment of atherosclerosis or cardiovascular risk factors, mainly statins but also angiotensin converting enzyme (ACE) inhibitors or angiotensin receptor AT1 antagonists [8,134,138] show pleiotropic antioxidant effects that might have contributed, at least in part, to the beneficial effects of these drugs on the treatment of atherothrombosis. Thus, atorvastatin acutely inhibits platelet Nox2, platelet isoprostanes and thromboxane A_2_ production and this leads to decreased oxidative stress and platelet activation [138]. Moreover, we and others have previously observed antioxidant capacity and inhibition of NADPH oxidase activation by statins in preclinical models [139,140]. Regarding the renin angiotensin system, it is well accepted that Angiotensin II, mainly acting on AT1 receptors, is one of the most important stimulus for oxidative stress both locally and systemically, through the activation of the NADPH oxidase among other mechanisms [5,14,16,28,29,30,141]. In fact, many studies have shown beneficial effects of ACE and renin inhibitors and AT1 antagonists on oxidative stress in different cardiovascular diseases (reviewed in detail [142,143]). It should be noted that recent evidence demonstrates that the selective AT2 receptor agonist Compound 21 decreases oxidative stress and atherosclerosis in an experimental model of diabetes-associated atherosclerosis [144] and might open new avenues for pharmacological treatment of atherothrombosis. Other drugs such as calcium channel blockers can also have potential antioxidant activities in the context of atherothrombosis [141,145]. However, only interventional well-controlled clinical trials with specific ROS inhibitors or supplement antioxidants will unequivocally confirm the role of ROS in atherothrombosis and the potential beneficial effects of these therapeutic approaches in patients at risk or having cardiovascular events.

**Figure 3:**
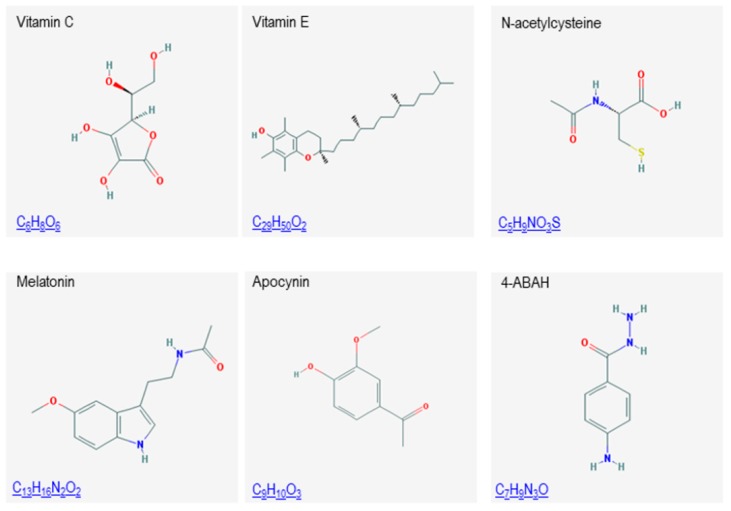
Chemical structures of different antioxidant compounds.

As mentioned above, animal models have provided important mechanistic information about the role of ROS and ROS-producing enzymes in atherothrombosis [24,25]. More importantly, preclinical animal models have set up the bases for a possible therapeutic effect of antioxidants in the clinic and therefore, although this review has focused on human studies, this aspect will be revised here in more detail. A general antioxidant melatonin (Figure 3) has been recently proposed as a potential agent for prevention of AAA [146]. More specific inhibitors of ROS have also been tested. Thus, administration of apocynin (antioxidant with some abilities to inhibit NADPH oxidase) attenuates experimental AAA formation and atherosclerosis progression [147,148]. It has also been shown that MPO inhibitor 4-amino benzoic acid hydrazide (4-ABAH) decreased vascular oxidative stress, consecutively improved endothelial function and significantly reduced atherosclerotic plaque development [149]. Very recently, MPO gene deletion attenuates experimental AAA formation [150]. Moreover, oral administration of taurine, an amino acid known to react rapidly with MPO-generated oxidants such as HOCl, also prevented AAA formation, reducing aortic peroxidase activity and aortic protein-bound dityrosine (diTyr) levels [150]. Treatment with the iron chelator desferrioxamine decreases lesion iron concentrations and inhibits atherosclerotic lesion development in the cholesterol-fed rabbit [151]. Iron restriction reduced the incidence of AAA formation with attenuation of oxidative stress and inflammation [62].

Raising HDLc using both genetic and direct infusion models similarly show global anti-atherosclerotic functions of HDL [152,153,154,155]. Similarly, treatment with HDL or fenofibrate inhibits experimental AAA formation and progression [156,157]. ApoA-I or ApoA-1 mimetics reduced or regressed atherosclerosis in animals, altering HDL function (e.g., inhibiting LDL oxidation) without changing HDLc mass [158,159]. Proposed mechanisms include accelerating HDL-mediated cholesterol efflux/reverse cholesterol transport and enhancing HDL’s anti-oxidant/anti-inflammatory properties [160]. In advanced aortic root atherosclerotic plaques of apolipoprotein E-deficient mice, native ApoA-I injections led to a significant decreases in lipid content, macrophage number, and an increase in collagen content; in contrast, oxidized human ApoA-I failed to mediate these changes [161]. Interestingly, modulation of HDL functionality by either ApoA1 mimetic D4F or PON1 overexpression decreased AAA formation in mice [95,117,162]. To add to this, PON1 overexpression in ApoE-KO mice displayed smaller atherosclerotic lesions as compared with control mice [163].

In relation to antioxidants, overexpression of catalase suppresses oxLDL-induced aortic smooth muscle cell death [164]. Atherosclerotic mice overexpressing catalase had smaller and relatively early stages of vascular lesions [165]. More recently, mitochondrial oxidative stress was successfully suppressed by catalase overexpression in mitochondria of macrophages or lesional myeloid cells of ApoE^−/−^ mice, and this led to a significant reduction in the aortic root lesional area [166,167]. In addition, catalase overexpression in aortic smooth muscle cells prevented pathological mechanical changes underlying AAA formation [168,169]. In addition, hemoxygenase-1 deficiency aggravates Angiotesin-II induced aortic aneurysms in the ApoE^−/−^ model [170].

## 6. Conclusions

Atherothrombosis is a very complex pathology that involves, among many other processes, lipid deposition, oxidative stress, inflammatory cell recruitment and platelet activation. Excessive ROS and oxidative stress (likely arising from both increased ROS generation from NADPH oxidase, MPO and iron, and decreased antioxidant systems from PON1 or catalase, among others), play an important role in the initial phases of the disease by inducing endothelial dysfunction (i.e., impaired NO-dependent vasodilation and increased endothelial activation) and by facilitating oxidation of LDL and HDL. In the more advanced stages, RBC-derived iron-rich heme group and leukocyte- and platelet-derived oxidants perpetuate the inflammatory process and eventually participate in the rupture of the arterial wall with subsequent platelet aggregation and thrombus formation. Findings from animal models and clinical studies prompted researchers to find oxidative stress markers in tissues and plasma of patients with atherothrombosis. However, although some of these oxidative stress markers predict increased CV risk, none of them have yet been incorporated into clinical practice. Notably, the concept of specificity does not imply their potential use as diagnostic biomarkers in the clinical setting as pathological biomarkers are not specific to a disease, but rather reflect a biological activity associated with pathology. In this respect, oxidative stress is underlying several different diseases and therefore we could observe modified levels of oxidative stress markers in different pathologies, not only in CVD.

We have focused this review on markers of oxidative stress associated with lipid/lipoproteins as they are the main drivers of vascular pathology, but it is important to note that oxidized lipoproteins could only be a consequence of increased oxidative stress and just reflect vascular disease without clearly proving their implication in promoting atherothrombosis. In fact, findings in patients treated with different antioxidant therapies are not conclusive despite the overwhelming information on the causative role of ROS in animal models of atherosclerosis and aneurysms. Similarly, we have reviewed the data on antioxidants as they have been studied more globally probably due to the higher stability and easier methodology used to address these questions. In this respect, we envision that novel methodological approaches (e.g., mass-spectrometry) will help to test more specifically the contribution of oxidative stress markers in the mechanisms of human atherothrombosis. In fact, these more specific markers of oxidative stress as surrogate prognostic/therapeutic markers could also potentially give interesting information at the clinical level. Finally, further studies, with more specific ROS inhibitors or antioxidants and carefully designed clinical trials, will probably shed light on the clinical benefits of targeting oxidative stress in CVD and its risk factors.
